# Controlling the impact of the managed honeybee on wild bees in protected areas

**DOI:** 10.1038/s41598-018-27591-y

**Published:** 2018-06-18

**Authors:** Mickaël Henry, Guy Rodet

**Affiliations:** INRA, UR406 Abeilles et Environnement, Avignon, F-84000 France

## Abstract

In recent years, conservation biologists have raised awareness about the risk of ecological interference between massively introduced managed honeybees and the native wild bee fauna in protected natural areas. In this study, we surveyed wild bees and quantified their nectar and pollen foraging success in a rosemary Mediterranean scrubland in southern France, under different conditions of apiary size and proximity. We found that high-density beekeeping triggers foraging competition which depresses not only the occurrence (−55%) and nectar foraging success (−50%) of local wild bees but also nectar (−44%) and pollen (−36%) harvesting by the honeybees themselves. Overall, those competition effects spanned distances of 600–1.100 m around apiaries, i.e. covering 1.1–3.8km^2^ areas. Regardless the considered competition criterion, setting distance thresholds among apiaries appeared more tractable than setting colony density thresholds for beekeeping regulation. Moreover, the intraspecific competition among the honeybees has practical implications for beekeepers. It shows that the local carrying capacity has been exceeded and raises concerns for honey yields and colony sustainability. It also offers an effective ecological criterion for pragmatic decision-making whenever conservation practitioners envision progressively reducing beekeeping in protected areas. Although specific to the studied area, the recommendations provided here may help raise consciousness about the threat high-density beekeeping may pose to local nature conservation initiatives, especially in areas with sensitive or endangered plant or bee species such as small oceanic islands with high levels of endemism.

## Introduction

In spite of the well-established environmental threats and economic difficulties affecting beekeeping sustainability, the amount of commercial honeybee (*Apis mellifera* L.) colonies has globally increased by 45% over the last half century, which denotes the rise of beekeeping industry in the era of economic globalization and growing worldwide human population^1^. In the meantime, seasonal migration of beehives (also called transhumance) has become a common practice in professional beekeeping where intensive agricultural systems have reduced floral resources and honey yields^2,3^. Often, these landscapes can no longer sustain apiaries all year round. Instead, beekeepers periodically move their hives into protected natural areas, with potential consequences for the integrity of the native pollinators’ interaction networks^4–6^.

A growing body of literature reports examples of massively introduced honeybees having detrimental effects on the abundance and diversity of local wild bees^4,7,8^. However, the findings are often inconsistent between different studies because the underlying ecological processes are not well understood and obviously depend on the local situation, including region^9^, habitat type and season^5,10–12^, or the degree of dietary specialisation in the wild bee population^13^. In the absence of clear evidence of the underlying ecological processes, and without specific guideline or legislation on this issue, protected land managers remain unaware of the potential threat high-density beekeeping poses to their nature conservation efforts. Uncertainty also fuels the debate among bee biologists^14–16^.

There are two main ecological processes by which massively introduced managed honeybees may compete with local wild bee populations and affect their spatial patterns of occurrence^4^. In *interference* competition^17^, the superior competitor directly deters others by physical interference. Interference competition, also termed interspecific displacement, is seldom reported to be the main driver of species occurrence in foraging bees^18^. Aggressive interaction may also be more often triggered by wild bees than honeybees^19^. In *exploitative* competition, the superior competitor indirectly alters other competitors’ fitness or abundance by monopolizing and depleting available resources. Although exploitative competition is expected to be the most common driver of species occurrence through space and time in the context of competition among foraging bees^20^, it has hardly ever been evidenced as such because it requires fine assessments of nectar and pollen resource availability. Instead, most studies have investigated side effects of honeybee-induced competition through the study of wild bee flower visitation rates^21^, body size^22^ or reproductive success^19,23^. Some studies have also reported long term wild bee declines and pollination network deficiencies that are possibly concomitant with the introduction of beekeeping in protected natural areas or other sensitive environments such as small oceanic islands with high levels of endemism^24–26^. In such large-scale correlative studies, it is however difficult to firmly establish the possible contribution of honeybee-induced competition.

In this study, conducted in a protected area exploited by managed honeybees, we first report evidence of wild bee occurrence patterns that are consistent with competition with the introduced honeybees. By “bee occurrence” we mean foraging intensity expressed as flower visitation rate. Second, we show that this pattern is mediated by exploitative competition for floral resources, based on measurements of harvested material in honeybee and wild bee nectar crops and pollen sacs. These estimates of foraging success reflect both interspecies competition between managed honeybees and wild bees inhabiting the protected area, and intraspecific competition among the honeybees themselves. Finally, we provide a rationale for guiding wild pollinator conservation policy in protected areas; we identify operational metrics and thresholds to guide decision-making by conservationists concerned by the impact of beekeeping on wild bee populations.

Sampling was carried out in a 5,700 ha area of Mediterranean scrubland, the Côte Bleue coastal area, southern France, of which 3,400 ha have protection status under Europe’s Natura-2000 programme. During the rosemary (*Rosemarinus officinalis* L., Lamiaceae) blooming period (c. 1 month, in March-April), up to 830 honeybee colonies are deposited in 28 apiaries – a density of >14 colonies.km^−2^. For comparison, the average national density is 2.5 colonies.km^−2^ in France^27^. During the 2015 and 2016 rosemary honey flows, a total of 180 honeybee and wild bee samples were collected at 60 sampling sites chosen in such a way as to cover the broadest possible range of distances from apiaries (850 ± 830 m (s.d.), range = [10 m, 4000 m]) and of colony density scores (128 ± 78 colonies, range = [12, 287]; see Methods for the density computation). Distance and density scores were analysed separately in relation to wild bee occurrence and foraging success, because land managers may use either colony distance or density metrics to regulate beekeeping, e.g. by setting distance or density thresholds. Wild bee occurrence was assessed as the number of foraging individuals counted on standardized amounts of flowering rosemary shrubs. The nectar and pollen foraging success was assessed in both wild bees and the honeybee. A nectar foraging success index was derived from measurements of harvested nectar in bees’ crop, collected with capillary glass tubes after applying a gentle pressure on their abdomen. The pollen foraging success index was obtained from measurements of pollen loads in their pollen collection apparatus.

## Results and Discussion

Overall, our results indicate that the wild bee occurrence pattern in the study area is compatible with competitive exclusion by managed honeybees. As a first striking feature, the study revealed that median honeybee occurrence was 15.3 and 12.9 times greater than that of wild bees in 2015 and 2016, respectively (58.5 *vs*. 3.8 and 78.8 *vs*. 6.1 visits per 100 units of rosemary flowering volume, respectively, see Methods). Those ratio cover more than an order of magnitude, mirroring the findings of other studies in mass-flowering areas where there is migratory beekeeping^5,28,29^. Most importantly, the wild bee occurrence rate was affected by the presence of managed bees. It decreased significantly with closeness to an apiary (Table 1, Supplementary Fig. S1) and with increasing honeybee colony density (Supplementary Table S1). In both cases, however, the pattern only emerged after a one-year time lag. For instance, median wild bee occurrence values dropped from 10.4 individuals at 1 km or more from the nearest apiary down to 4.4 individuals at shorter distances. This suggests that shorter distances to, or higher densities of, honeybee colonies cause wild bees to disperse further afield and/or depress their fitness^23^, with consequences for the next generation’s local occurrence rate. Indeed, as most wild bee species are solitary and univoltine, i.e. with a single generation per year, their current occurrence pattern typically depends on the previous year’s nesting conditions, particularly the floral resources available for provisioning nests^30,31^.

**Table 1:** Effect of increasing distance to the nearest apiary on bee occurrence and foraging success.

Bee occurrence and foraging response variables*	Sample size (Nb of sites)	Intercept	Estimates^†^	Statistics	P-value (effect sign)	AIC weight (*ω*) ^‡^
**Wild bees**						
Wild bee foraging occurrence, inter-annual scale (Foraging intensity for 100 flowering volume units)	180 (60)	2.63 ± 0.22	0.46 ± 0.171	z = 2.71	**0.006 (+)**	78.4%
Wild bee foraging occurrence, annual scale (Foraging intensity for 100 flowering volume units)	180 (60)	2.68 ± 0.20	0.26 ± 0.13	z = 1.92	0.055	
Mean nectar foraging success (Standardized nectar crop content)	82 (35)	23.67 ± 2.61	8.43 ± 3.85	t = 2.19	**0.033 (+)**	>99%
Mean pollen foraging success (Pollen load score)	78 (39)	29.16 ± 4.50	3.10 ± 3.80	t = 0.82	0.42	
Body size, inter-annual scale (Body length, mm)	220 (44)	12.84 ± 0.36	1.02 ± 0.37	t = 2.75	**0.006 (+)**	>99%
Body size, annual scale (Body length, mm)	220 (44)	12.83 ± 0.26	1.18 ± 0.37	t = 3.22	**0.001 (+)**	>99%
**Honeybees**						
Mean nectar foraging success (Nectar crop content, μl)	144 (49)	4.37 ± 0.54	1.64 ± 0.32	t = 5.17	**<0.001 (+)**	>99%
Mean pollen foraging success (Pollen load score)	106 (44)	1.58 ± 0.29	0.66 ± 0.22	t = 3.02	**0.004 (+)**	>99%

Wild bee occurrence in foraging surveys is better explained by the previous year’s apiary distances (inter-annual scale) than by current year distances (annual scale). Analogous statistics for colony density effects are shown in Supplementary Table S1.

^*^All models are LMMs, except wild bee foraging occurrence: Zero-Inflated GLMM (negative-binomial family distribution and log-link function); ^†^Estimates stand for changes per apiary distance unit (km, with log-correction in wild bees); ^‡^AIC weight of evidence in favour of the apiary distance effect being a better predictor than the colony density effect. The AIC weight *ω* is shown only when at least one of the two candidate predictors has a significant effect (see Supplementary Table S1 for the colony density effect).

Exploitative competition, rather than interference competition, is the most obvious underlying process for this effect on wild bee occurrence. First, despite many thousand observations of foraging bees in the field, aggressive interactions between foraging honeybees and wild bees have seldom been witnessed (M.H. and G.R., personal observations), just as in other studies on the subject^18,19^. Secondly, the large-bodied bees (e.g. *Anthophora*, *Bombus* or *Xylocopa* species, Supplementary Table S2), which are more likely to physically outcompete honeybees, were in fact more prone to competitive exclusion, as they were found farther away from the apiaries (Table 1, Supplementary Fig. S2). Although rather weak (12% body size decrease around apiaries), this finding is consistent with the exploitative competition hypothesis. Large-bodied bees need more pollen and nectar, and will therefore suffer the effects of exploitative competition before small-bodied ones^6^. Larger bees are also more mobile and can easily disperse away from apiaries to forage and nest in low-competition areas^32,33^. Finally, exploitative competition was directly evidenced by the decrease in nectar foraging success with decreasing distance from apiaries, in both honeybees and wild bees (Table 1, Supplementary Fig. S1). This was revealed by nectar crop measurements carefully controlled for body size variations (see Methods). Exploitative competition for pollen, however, was only evidenced at the intraspecific level. Honeybee pollen foraging success was significantly lower where apiaries were closer (Table 1, Supplementary Fig. S1), while no significant pattern emerged in wild bees. Although speculative at this point, it is possible that the spatial rearrangement of foraging wild bees along the apiary distance gradient has successfully achieved an Ideal Free Distribution (*sensu*^34^) for pollen harvesting, whereby the lower competitors adjust their distribution so as to balance costs and benefits of foraging and to equalize foraging success^35^.

**Figure 1:**
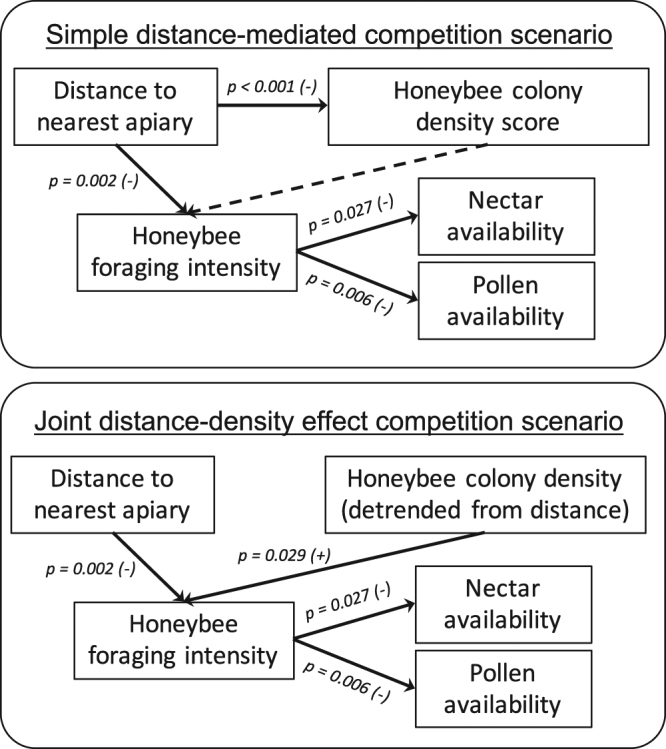
Reconstruction of the most plausible causal chain scenario behind honeybee intraspecific competition. Arrows indicate causal links among variables. *P*-values and signs stand for link significance and effect direction. In a first tentative path model scenario, apiary distance was positioned as the only proximal driver of honeybee occurrence (foraging intensity) in sampling sites, affecting in turn nectar and pollen availability in flowers. However, a significant effect of colony density scores, conditional on distance, was detected in the causal chain (dashed arrow, see Supplementary Table S5 for detailed statistics), making the conditional independence requirements close to being rejected (*d*-separation test of deviation from conditional independences, *C* = 19.22, *df* = 12, *P* = 0.083). A joint distance-density effect path model was also computed, whereby apiary distance and colony density jointly influenced honeybee foraging intensity (see Methods for the computation of detrended colony density). The joint distance-density effect scenario satisfactorily met the conditional independence requirements (*C* = 8.75, *df* = 10, *P* = 0.56) and was far better supported than the distance scenario, given the much greater AICc weight of evidence (*ω* > 99% in favour of the joint effect scenario, Supplementary Table S5).

Still, further data on pollen and nectar availability were needed to disentangle the effect of intraspecific competition from a possible behavioural trade-off, in honeybees, between distance and harvest. We therefore collected additional field data (see Methods) on nectar and pollen availability in rosemary flowers and performed a confirmatory path analysis^36,37^ to reconstruct the most plausible chain of causal links with honeybee metrics (Fig. 1). The best causal scenario behind the honeybee intraspecific competition involves apiary distance and colony density as joint drivers of honeybee foraging intensity. Greater honeybee foraging intensity led in turn to lower pollen and nectar availability in rosemary flowers. This result validates the hypothesis of intraspecific exploitative competition among honeybees for pollen and nectar where colony density is high. Distance to nearest apiary was still the key driver of competition in the studied scrubland (see AIC weights in Table 1) and should therefore take precedence over density score as a criterion for regulating beekeeping.

**Figure 2:**
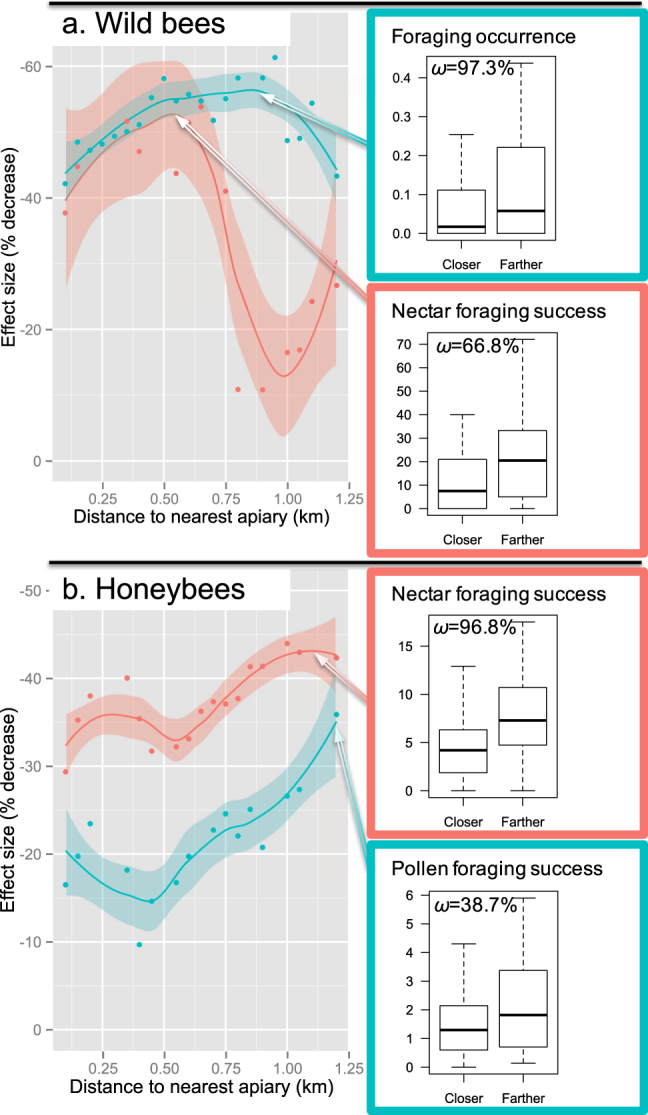
Beekeeping-induced competition as a function of distance from nearest apiary in (**a**) wild bees and (**b**) honeybees. The competition effect size (percentage decrease in foraging intensity and foraging success close to apiaries) was recomputed by varying the distance between closer and farther sites, from the first (150 m) to the third (1,200 m) quartiles of distances covered in the study. Trends are depicted by LOESS local regression fits and 95% confidence envelopes (solid lines and coloured areas respectively). For each competition metric, the panels on the right show the distribution of values (quartile boxes) for sites located closer to *vs*. farther away from the nearest apiary. The AIC weight *ω* gives the probability that competition is better accounted for by a two-step threshold effect model (closer-*vs.-*farther binary distance variable) rather than a progressive effect model (continuous distance variable). Thresholds emerged (*ω* >> 50%) at 600 m for wild bee nectar foraging success, at 900 m for wild bee occurrence and at 1100 m for honeybee nectar foraging success. The progressive effect model was better supported for honeybee pollen foraging success (*ω* << 50%) as effect size did not peak but steadily increased until 1200 m. Effect sizes for wild bee occurrence were based on the inter-annual scale (Table 1). See Supplementary Table S6 for detailed data and sample sizes at each distance class and AIC model selection statistics.

Practical thresholds emerged for apiary distance vs. competition effect size, paving the way for pragmatic decision-making by managers of protected land who are concerned with wild bee conservation (Fig. 2). We have expressed competition effect size (%) as the percentage changes in bee foraging success and occurrence with distance from nearest apiary. Effect sizes were recursively recomputed by adjusting the distance limit between closer and more distant sites, from the first (150 m) to the third (1,200 m) quartiles of distances covered by the study (Fig. 2). We detected marked effect size peaks which showed that competition was highest within a certain distance from an apiary and relaxed beyond that distance. The competition effect was operative (*i*) 600 m away from the apiary with a 50% decrease in wild bee nectar foraging success, (*ii*) 900 m away with a 55% decrease in wild bee occurrence, (*iii*) 1,100 m away with a 44% decrease in honeybee nectar foraging success and (*iv*) 1,200 m away or more with a 36% decrease in honeybee pollen foraging success (Fig. 2). The honeybee distance thresholds fell within the median foraging ranges of 1–2 km usually reported in the literature^38–40^. It also fits distances at which detrimental effects of apiaries have been reported on native bumblebee foraging behaviour (until 1,200 m in^41^).

We argue that protected land managers could use honeybee intraspecific competition as a criterion to guide their regulation of mass-flowering resource exploitation by beekeepers. Competition effect size measured in wild bees peaked at shorter distances from apiaries (600–900 m, Fig. 2) than honeybee intraspecific competition (Nectar: 1.1 km, pollen: >1.2 km, Fig. 2). Therefore, any buffer distance rule derived from the latter metrics will be more conservative regarding wild bee protection. It will also inform managers about the carrying capacity of the area and help optimize beekeeping honey flows. For instance, in the Mediterranean scrubland area we studied, with an average apiary size of 30.9 ± 21.8 (s.d.) colonies, intraspecific competition for nectar is theoretically relaxed beyond the effect size peak distance of 1.1 km. Thus the area theoretically reaches saturation with a distance of 1.1×2 = 2.2 km between neighbouring apiaries, i.e. ~1 apiary per 3.8 km^2^ (that is 0.26 apiary/km^2^). Managers wishing to allocate half an area to wild bee conservation under relaxed competition will then need an apiary load twice as low, i.e. ~1 apiary per 7.6km^2^ (that is 0.13 apiary/km^2^ or 3.1 km spacing between apiaries).

Overall, those distance-based recommendations do not return honeybee colony densities that are fundamentally different from others found in literature. Considering our average apiary size of about 30 colonies, the “saturation” (0.26 apiary/km^2^) and “half-saturation” (0.13 apiary/km^2^) apiary loads are equivalent to 7.8 and 3.9 colonies/km^2^, respectively. Interestingly, the latter value, which stands for a 50% land-sharing between honey production and wild bee conservation, is only slightly more permissive than previous recommendations (3.1 colonies/km^2^ in^42^ or 3.5 colonies/km^2^ in^6^). But most importantly, the distance-based thresholds we suggest herein might help inform the recent debate on the effectiveness of beekeeping regulation in protected areas with respect to honeybee foraging range^14,15^. Given the high mobility of foraging honeybees, protected areas may be theoretically exploited from off-site apiaries located up to 10 km away, potentially making local regulation rules inefficient^14^. On the other hand, such extreme foraging ranges are thought to be uncommon, and competition effects is rather expected to range around the mean honeybee foraging distances, i.e. about 1 km away from apiaries^15^. Here, we provide empirical support to the view that local beekeeping regulation will indeed benefit wild bee conservation in protected areas. We based our reasoning on functional aspects of foraging ecology, rather than on any rough guess of the actual honeybee foraging range.

Managers of protected land should apply the distance-based thresholds only where natural mass-flowering resources are over-exploited, with a view to gradually reducing existing beekeeping pressure. Managed honeybees should not be introduced into pristine areas or areas with sensitive or endangered plant or bee species^4^, such as in small oceanic islands with high levels of endemism^24–26,43^. In continental environments, managers of protected areas may also constrain the location of apiaries by applying the distance-based regulation threshold around habitats or microsites of special conservation interest that are identified to host threatened or emblematic plant or pollinator species. Thresholds should be carefully re-evaluated for each situation concerned. In addition to threshold-based regulation, land managers could envision periodic break years to temporarily halt competition disturbance regime and boost resilience in wild bee populations.

Regulating colony density will also benefit honeybees themselves. Beekeepers may not perceive a substantial honey yield decrease under high-density management, owing to the colonies’ internal regulatory processes. But exploitative competition may constrain honeybee foragers’ lifespan and trigger a cascade of problems that eventually leads to colony weakening or collapse later in the season, long after the migration period has ended^3^.

The honeybee tends to take precedence over wild bees as a target species in conservation programs because for policy makers and land managers the honeybee is an emblematic pollinator species^7^. Some European countries, including France, have agro-environmental schemes that envision subsidising beekeepers to set up apiaries in natural areas. We believe these agro-environmental schemes should, on the contrary, reward beekeepers who make joint efforts with farmers to maintain their apiaries in agro-ecosystems all year round. Protected land managers and beekeepers should realise that mass-flowering resources in natural areas are shared resource systems. If beekeepers exploit them independently, according to their own self-interest, they have a high chance of acting contrary to the common good of all users, others beekeepers and wild bees, by depleting or spoiling that resource through their cumulative actions. This is called *the tragedy of the commons*^44^.

## Methods

The hypotheses of altered wild bee occurrence and depressed foraging success around apiaries were investigated by means of honeybee and wild bee occurrence surveys and foraging success assessment in a range of sampling sites located at different distances from apiaries in a protected natural area.

### Study area and sampling design

The Côte Bleue study area is a 5.700-ha protected Mediterranean scrubland, dominated by Kermes oak *Quercus coccifera* and Rosemary *Rosmarinus officinalis*. The area is managed by the French coastal protection agency (Conservatoire National du Littoral) in partnership with the French National Forest Office (Office national des forêts, ONF). Each year, ONF allocates 28 sites in the Côte Bleue to accredited beekeepers so that the location and size of each apiary is thoroughly registered. Those apiary registration data were systematically ground-truthed at each study year. Beekeepers typically set up apiaries for rosemary honey production during ca. 1 month early in the season, in between Mars and April.

During the 2015 and 2016 rosemary blooms, a total of 180 wild bee field samples were carried out in 60 structurally similar scrubland sampling sites chosen so as to cover broad gradients of beekeeping intensity in terms of nearest apiary distance and size. The sampling design was however constrained by inter-annual fluctuations in the presence of apiaries. We varied accordingly sampling site locations between years (Supplementary Fig. S3) in order to maintain a balanced site allocation across beekeeping gradients. Data analysis was therefore based on a generalised mixed model framework to account for the resulting spatial and temporal nested design (see *Data Analysis*).

Sampling site selection was first driven by apiary location, and then by local rosemary flowering cover. The area was subdivided into four main contiguous sectors with different access trails. In each sector, a first series of sites were chosen for their proximity to the main apiaries, i.e. a few tens of meters away, corresponding to the smallest foraging range usually reported in literature for wild bees^45^. Then, a second series of sites were located as far as possible from apiaries (1 to 4 km). Finally, additional sites were chosen at intermediary positions on the way, so that the overall design covered a wide range of distances (850 m ± 830 m (s.d.)) with balanced site numbers among sectors. Sites were defined as an area of 50 m in diameter with a widespread, uniform, rosemary distribution – totalling on average 10% to 15% of soil cover. Rosemary is by far the dominant flowering resource throughout the scrubland, and particularly during the beekeeping migration period when secondary resources (mainly *Thymus vulgaris*, *Cistus albidus*, *Cistus salviifolius*, *Helianthemum marifolium*, *Reseda phyteuma*), have not bloomed yet. We still controlled for food resource conditions by avoiding sites with conspicuous or unusually dense patches of secondary resources. Accordingly, only 3.5% of sampled bees were captured on the secondary floral resources, which we considered to be too restrictive to deserve specific analyses.

All sampling sites within a given sector were typically processed during the same half-day. No sample was performed under rainy, windy or cold (<12°C) weather. All sectors were therefore covered in 2-days sessions. The rosemary blooming period was covered with a total of six sessions each year. Depending on local phenological fluctuations, individual sites were visited on average 3.0 ± 1.6 (s.d.) times. During a given session, we randomized the sector visit order so that sample locations were independent from time of the day (varying from 9:00 to 18:00, solar time). Finally, we assumed that our sampling design was poorly affected by possibly undetected large apiaries located outside of the boundaries of the protected area. The area is bordering the Mediterranean sea on the south part, and is surrounded by a large (155 km^2^) water body, the Pond of Berre, on its West and Northern parts. The immediately adjacent lands on the North and East parts of the area are densely urbanised and are not suitable for professional apiaries.

### Wild bee occurrence

Field samples consisted in the joint assessment of the local rosemary floral resource availability and of the wild bee occurrence on those flowers (foraging intensity expressed as flower visitation rate). Fifteen flowering rosemary shrubs were carefully inspected, starting from the largest one in the site, and then moving step by step to the nearest neighbours. Contiguous shrubs with undistinguishable, coalescing crowns were treated as a single individual. Shrub flowering volume estimates were derived from the three shrub crown dimensions (length, width and height) rounded to the nearest 50 cm, which we defined as the smallest tractable volume dimension unit. If necessary, shrubs with irregular shapes were sized in two or more steps. Shrub flowering volumes were further weighed by a floribondity multiplicative coefficient, reflecting the percentage (±10%) of open flowers relatively to the expected maximal number of flowers branches may actually bear (up to 80 flowers per 20 cm). For instance, if a shrub is visually estimated to be at 50% of its flowering potential, its crown volume is then corrected to half its actual size. Finally, we tallied foraging honeybees and wild bees while inspecting shrubs, and computed the corresponding numbers of bees per unit of flowering rosemary volume to serve as a foraging intensity measurement. The same two observers performed all those field estimates. Preliminary blind comparisons led to consistent and highly correlated estimates between observers. Nevertheless, an observer was kept unchanged throughout each sampling session to avoid biases. The bee survey routine included net captures for assessing wild bee and honeybee individual foraging success.

### Individual Foraging success

Foraging success was assessed in female wild bees and honeybees using nectar crop content and pollen load measurements. A variable transect walk (*sensu*^46^) was performed to collect all bees with nets. observers walked at moderate speed among flowering rosemary shrubs and collected bees during a minimum of 20 minutes.person, which was usually sufficient to capture at least 10 foraging honeybees. We did not constrain samples with a minimum number of wild bee captures because those were much less abundant. Captured individuals were maintained in a cooler at about 4°C to slow down their metabolism before being processed. Throughout the study, we favoured non-invasive methods. Once processed and identified to family or genus, wild bees were released right on the capture site. Only few specimens per morphotype were collected for subsequent identification to species. The resulting preliminary checklist for the study area is provided in Supplementary Table S2.

Nectar foraging success was assessed by measuring the nectar volume stored in their crop stomach at the time of capture, also termed field nectar load^47,48^. Bees were first narcotized for few seconds with CO_2_ (ProFlora U500 Cylinder, JBL GmbH & co, Neuhofen, Germany). A gentle dorso-ventral pressure was then applied on their abdomen until their crop nectar content was regurgitated. The extracted nectar was capillary-collected (10 μl micropipettes Ringcaps, Hirshmann Laborgeräte GmbH & co, Eberstadt, Germany) for volume measurement with an estimated ± 0.05 μl resolution. We then used a refractometer (REF108, Index Instruments Ltd., Cambridgeshire, England) to ascertain the presence of high concentrations of sugar. On very rare occasions, crop content was identified to be mostly water, with null or low (<5%) sugar concentrations. Bee water foragers were discarded from the nectar foraging databases. Finally, we measured (to the nearest mm) wild bee body length, from head to abdomen extremity, for allometric standardization. Indeed, the volume of nectar wild bees store in their crop is first and foremost dependent on their body size, which may vary by more than an order of magnitude depending on species. We then converted raw nectar volume data into size-specific nectar volumes, scaled on expected maximal field nectar loads given body length.

Maximal values of field nectar loads were singled out from each individual bee size class (body length rounded to nearest mm) with at least five non-null measurements. Those maximal field nectar loads were satisfactorily modelled as a function of wild bee body length (Supplementary Fig. S4) using a power law, following usual allometric scaling properties^49,50^. The nectar load (μl) was then standardized to the predicted maximal load for the considered wild bee body length (mm) using the expression:

$${\rm{Standardized}}\,{\rm{nectar}}\,{\rm{load}}={\rm{nectar}}\,{\rm{load}}/(0.005\times {\rm{Body}}\,{{\rm{Length}}}^{3.0618}).$$

The standardisation was successful to deliver nectar load data independent from bee size (linear model, n = 219, t = −0.316, p = 0.75).

Pollen foraging success was assessed by measuring pollen loads honeybees and wild bees had harvested in their pollen sacs, also called scopa or pollen-carrying apparatus, at the time of capture. For honeybees which compact pollen into well-defined pellets on their hind legs, pellet dimensions were measured to the nearest 0.1 mm with a vernier, and converted into a volume following the ellipsoid formula: Volume = (4/3) × π × length × width × height.

For wild bees, we resorted to a *fuzzy coding* approach to standardize pollen loads among bees of different size or with different scopa configurations (i.e. hind leg *vs*. ventral scopa). We reported a pollen load score indicating the estimated percentage (±10%) of expected maximal scopa load. Maximal scopa load was based on maximal observed pellet size for hind leg pellets (e.g. bumble bees), or on maximal scopa cover for ventral scopa (e.g. megachilid bees). The kleptoparasitic bees (e.g. *Nomada* species) that rely on pollen stored by their host, and then lack scopa, were discarded from the database. Likewise, a few bees carry pollen internally in their crop (*Hylaeus* species) and therefore could not be included in the analysis.

In the course of a foraging bout, honeybee foragers may be assigned a specialized foraging strategy for either pollen or nectar, or a mixed foraging strategy. Herein, the vast majority of captured bees displayed quantifiable amounts of both pollen and nectar. Therefore, we were unable to assign bees a consistent foraging category. All foraging individuals were considered indistinctly in foraging success analyses.

### Beekeeping metrics

Conservationists and land managers may regulate beekeeping in protected areas using threshold decision rules based on minimum colony distances or maximum colony densities. We therefore quantified beekeeping around sampling sites using (i) distance to nearest apiary and (ii) a spatially explicit colony density score, which incorporates both colony distances and densities throughout the study area. The spatially explicit density uses an ordinary inverse distance weighted interpolation, whereby the size of each apiary (number of colonies) in the study area is weighted inversely to its distance by a 1/*d*^2^ multiplicative coefficient, with *d* the distance (km) to the considered sampling site. Following previously suggested settings for honeybee foraging studies^51^, the coefficient was set to 1 for apiaries <1 km away, i.e. the approximate lower median foraging distance reported for honeybees^39^. The resulting density scores are actually the sums of the distance-weighted size of all apiaries in the area. Scores ranged from about 12 to 287 colonies, with an average of 128 ± 78 (s.d.). Most importantly, we also computed beekeeping distance and density metrics on an inter-annual basis. This was particularly relevant for wild bee occurrence data since the current demographic state of univoltine insect populations actually reveals nesting and reproductive success of the previous year.

Not surprisingly, apiary distances and colony densities around sampling sites were significantly and negatively correlated (annual scale: Pearson *r* = −0.58, *df* = 58, *P* < 0.001; inter-annual scale: Pearson *r* = −0.47, *df* = 58, *P* < 0.001), but each conveys information of specific relevance for land managers. They were therefore analysed separately in relation with bee occurrence and foraging success.

### Data Analysis

Bee foraging success and occurrence data were confronted to beekeeping metrics using (generalized) linear mixed effect models (G)LMMs. We accounted for the spatial dependency of data originating from the same site and from the same sector during a given year by specifying the corresponding variables (year, sector and site identity) as random grouping terms^29,52^. Analyses were performed with the R software for statistical computing, v. 3.1.0 (R Development Core Team 2014). LMMs and GLMMs were computed with the *lmer* and *glmmADMB* packages, respectively. Power analyses were performed and upgraded in the course of the study to ensure the adequate statistical resolution of the experimental design (Supplementary Table S3). Power was assessed using the *pwr.f2.test* function of the *pwr* package, specially suited for generalized linear models. We targeted a 90% statistical power for detecting a medium effect size (*sensu*^53^) at a significance level α = 0.05. To achieve the desired power, we surveyed wild bees at all the 12 sampling sessions, and assessed nectar and pollen at ten and eight sessions, respectively, evenly allocated between study years. Model residuals diagnostic plots were inspected to ensure residual normality and homogeneity requirements were satisfactorily met.

Wild bee foraging intensity models delivered unsuitable residual distributions due to the numerous zeros in the dataset. We therefore recomputed a zero-inflated model (ZI-GLMM) using a negative-binomial family distribution with a log-link function, which is well suited for count data that are subject to overdispersion, such as individual surveys^54^.

Likewise, individual foraging success LMMs were not satisfactory regarding residual normality requirements. To restore residual normality and homogeneity, we averaged individual data per sampling site and session. We therefore specified a variance weighting term to properly weight data by the number of individuals the averaged foraging success is actually based on. Finally, a log-correction of apiary distances was necessary to further reduce residual variance in wild bee models, which are based on fewer data points than honeybees.

We were also concerned that foraging success data could be influenced by the time of the day, especially for pollen, which is offered at once at the onset of anthesis in the morning. Nectar secretion, on the contrary, continues throughout the day. Regardless the expected pattern, we systematically tested the time effect prior to analyses. No significant temporal variation was recorded in wild bee foraging intensity (ZI-GLMM, *n* = 180, *t* = −1.35, *P* = 0.18) and nectar foraging success (LMM, *n* = 82, *t* = 1.21, *P* = 0.23) or in the honeybee nectar foraging success (*n* = 144, *t* = −1.91, *P* = 0.059). On the contrary, pollen foraging success decreased significantly during the day in both wild bees (*n* = 91, *t* = −2.82, *P* = 0.007) and the honeybee (*n* = 129, *t* = −2.44, *P* = 0.017). However, we noticed that this temporal pattern was mostly explained by a depletion of pollen harvests toward the end of the day. Accordingly, we distinguished the late samples, carried out during the fourth time quartile (>16:00) from the main daytime samples collected earlier, during the first to third time quartiles. The temporal phase of the day (main-*vs*.-late samples) was then included in pollen models in interaction with the focus beekeeping metrics. As a precaution, on top of the complete models with temporal interaction, we computed simple models that focused on the one-way effects of beekeeping during the main sampling daytime only. The two approaches delivered identical conclusions regarding beekeeping effects. For the sake of simplicity, the one-way models are shown as a part of the core results (Table 1), and the complete temporal interaction models further detailed in the Supplementary Table S4.

### Confirmatory path analysis

As consistent beekeeping effects emerged on the honeybee foraging success metrics (see Results), we carried out complementary field measurements and a confirmatory path analysis^36^ to further support the intraspecific competition hypothesis. Those were intended (i) to disentangle the respective influence of colony densities *vs*. distances on honeybee foraging success and (ii) to find evidence of depressed pollen and nectar availability with increased honeybee density. Nectar and pollen resource availabilities were assessed during the 2016 rosemary blooming season. Available nectar was quantified in sampled sites by introducing tiny 1 μl micropipettes (ref. 0227726, CAMAG, Muttenz, Switzerland) inside rosemary flowers to reach the nectaries at the bottom of the corolla tube. The so-called *nectar standing crop*^6^ available to visiting insects was expressed as a cumulative nectar volume for 100 sampled flowers, considering two flowers per branch, two branches per individual rosemary shrub, and at least 20 sampled shrubs. Nectar samples were duplicated to ensure the sampling design was robust enough to deliver repeatable estimates.

Likewise, we sought for a simple index of rosemary pollen availability that could be readily acquired during the sampling routine. In rosemary flowers, sexual organs are positioned above the corolla entrance, so that pollen is deposited on the back of insects that insert head into the corolla for harvesting nectar. Therefore, captured honeybees are found with pollen deposits of various sizes on their thorax, depending on pollen availability on stamens. We attributed honeybees a pollen deposit score, analogous to the pollen load score in wild bee ventral scopa (see above), based on the proportion (±10%) of the thorax dorsal surface covered with pollen deposits. Pollen deposit scores were averaged among ten captured honeybees in a given site visit to serve as pollen availability index. Although rather rough, this surrogate of pollen availability was measurable on a routine basis. Contemporaneously, honeybee foraging intensity (amount of foraging honeybees per unit of flowering rosemary volume, see above) was recorded, with the underlying idea that it will influence the local nectar and pollen availabilities.

The confirmatory path analysis was aimed at unravelling the ecological processes driving rosemary nectar and pollen resource availability under beekeeping exploitation. We compared a *simple distance-mediated* competition scenario and a *distance-density joint effect* competition scenario, whereby honeybee foraging intensity was influenced either solely by distance to the nearest apiary, or jointly by the combined effects of apiary distance and colony density in the area. In the joint effect scenario, colony density scores were beforehand detrended from distance by extracting the corresponding residuals. Detrended colony density therefore discriminates high- from low-density areas, respectively with positive and negative residuals, while controlling for the nearest apiary distance. In both scenarios, we expected that foraging intensity would in turn depress local nectar and pollen resource availability.

Path analyses help reconstruct the most plausible chain of causal links in multivariate datasets by assessing conditional independences among indirectly linked variables^36,37^.

Two response variables may express colinearity if they are concomitantly influenced by the same explanatory variable. The two response variables are said conditionally independent if colinearity disappears when statistically controlling for the explanatory variable effect. Deviation from expected conditional independences was assessed using the *d*-separation test, specially suited for the linear (mixed) model frameworks^36^, in the *piecewiseSEM* R package. Conformingly to the previous analysis settings, we used LMMs to formalise the links of the path model scenarios connecting beekeeping metrics to honeybee foraging intensity and then nectar and pollen availabilities. In line with the pollen foraging success analyses, we controlled for the daily pollen depletion pattern by restricting pollen LMMs to the main daytime samples (first to third time quartiles). We finally computed the AICc (Akaikee information criterion AIC corrected for small samples) value of each candidate path model to compare them based on fit and complexity^37^, the lowest AIC indicating the most plausible scenario. Following usual information theoretic procedures^55,56^ we calculated the weight of evidence in favour of the best scenario, termed AIC weights *ω*.

### Data availability

The apiary location dataset analysed during the current study is not publicly available in order to protect the privacy of local beekeepers, but are graphically shown on Supplementary Fig. S3 and are available from the corresponding author on reasonable request. Detailed data in support of analyses shown in Fig. 1 (path analysis) and Fig. 2 (Threshold analysis) are available in Supplementary Tables S5 and S6, respectively.

## Electronic supplementary material

Supplementary Information

Supplementary Table S6
