# Supplementary material for: Controlling the impact of the managed honeybee on wild bees in protected areas
Source: Sci Rep. 2018 Jun 18;8:9308. doi: 10.1038/s41598-018-27591-y (PMC6006304; doi:10.1038/s41598-018-27591-y)
Supplement: Supplementary file 1 — Supplementary Information [file 41598_2018_27591_MOESM1_ESM.pdf]

Supplementary Information

Controlling the impact of the managed honeybee on wild bees in protected areas

Mickaël Henry* & Guy Rodet

INRA, UR406 Abeilles et Environnement, Avignon, F-84000, France.

* To whom correspondence should be addressed: mickael.henry@inra.fr

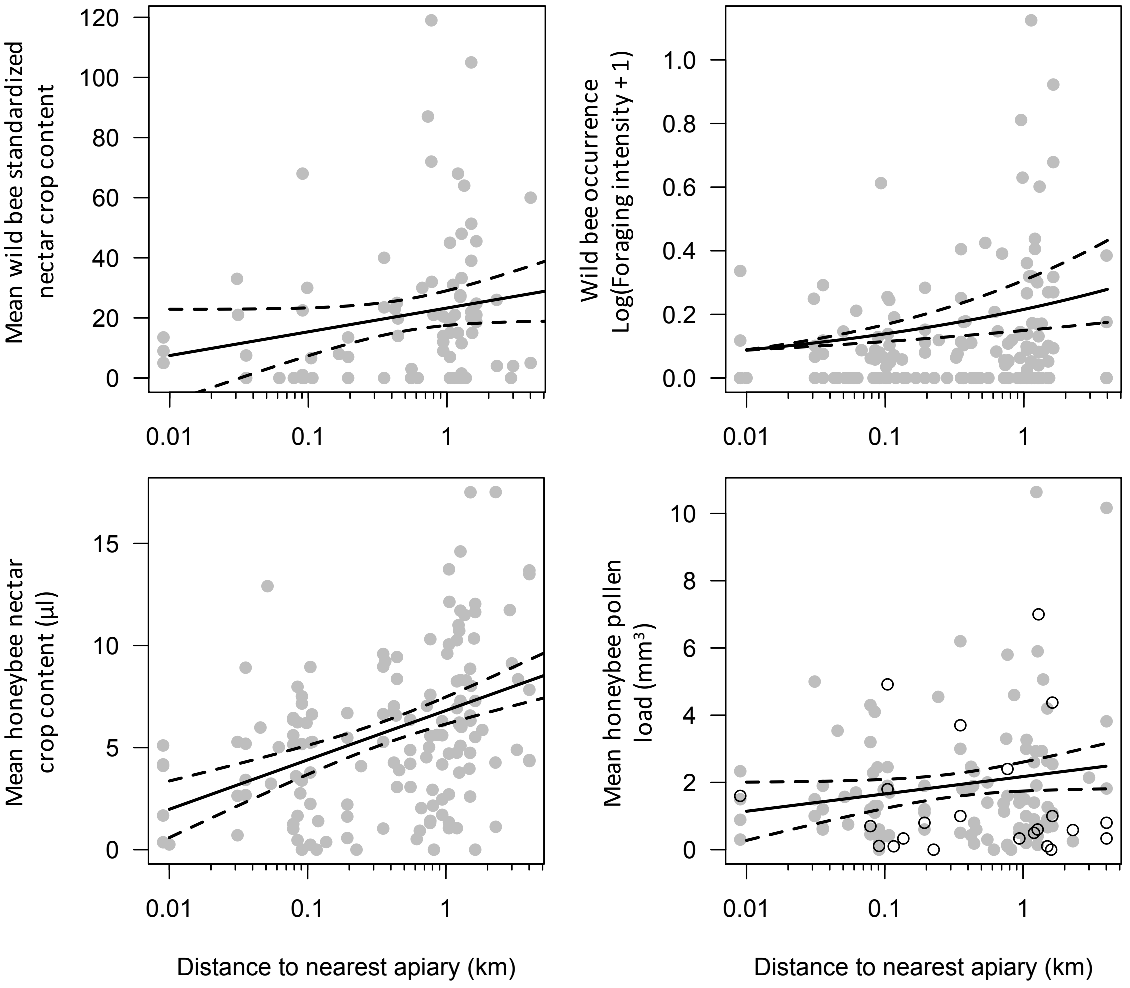

Supplementary Figure S1. Representation of the significant effects of nearest apiary distance on bee competition metrics. Distances were based on previous year apiary locations for wild bee occurrence, and on current year locations for other metrics. Solid and dotted lines stand for best fits and 95% confidence envelops, respectively. In honeybee pollen load, closed and open dots stand for main *vs*. late daytime samples, respectively, that were distinguished to control for temporal variations.

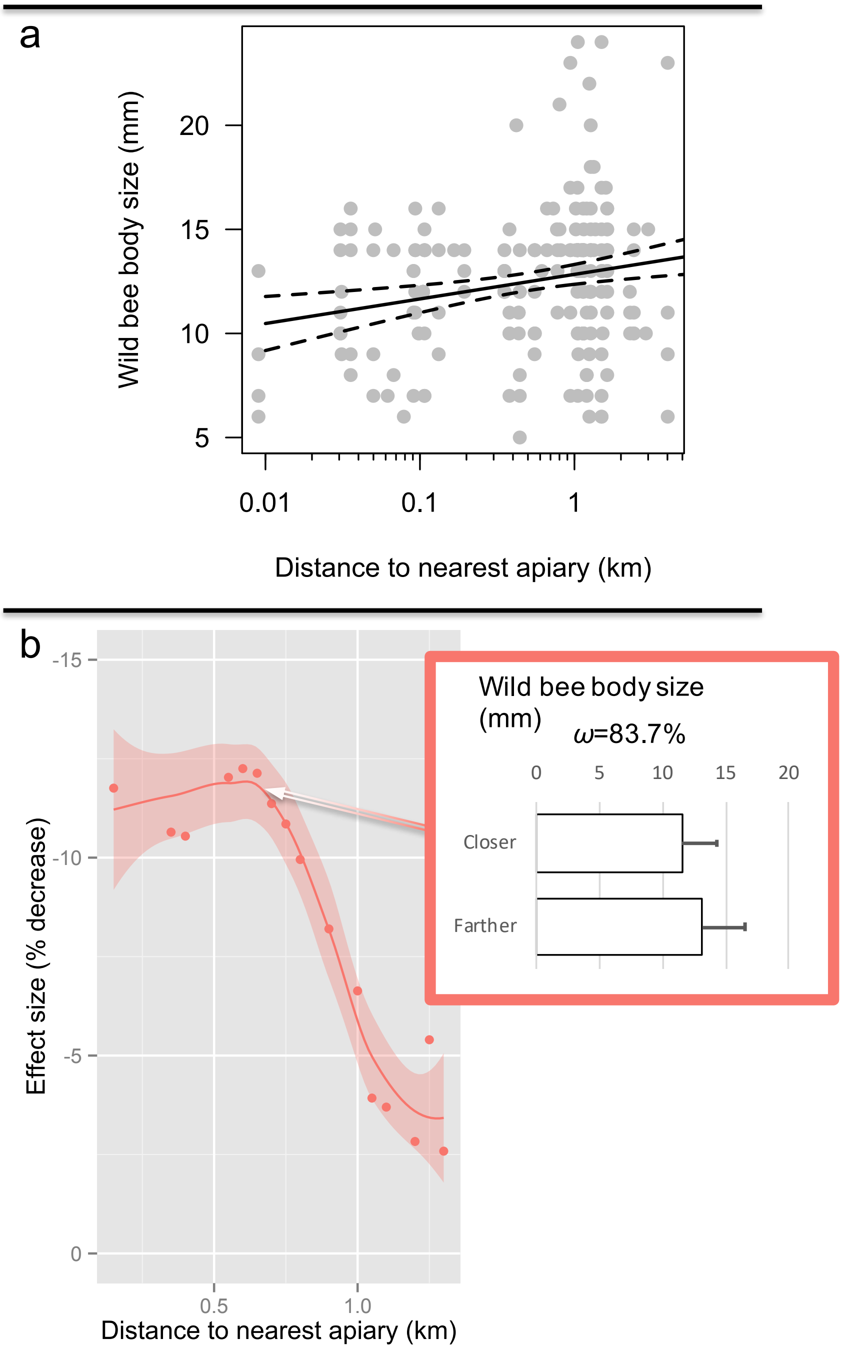

Supplementary Figure S2. Wild bee body size increase with distance to the nearest apiary. (a) Solid and dotted lines stand for best fit and 95% confidence envelop, respectively. See Supplementary Table S2 for bee species potentially covered by different body size ranges in the study area. (b) Competition effect size (percentage decrease in average body size close to apiaries) as a function of distance to apiaries, showing an emerging threshold at about 650m. The inset compares average (±s.d.) wild bee body size on both sides of the apiary distance threshold. Legend follows Fig. 2. See Supplementary Table S6 for detailed data and sample sizes at each distance class and AIC model selection statistics.

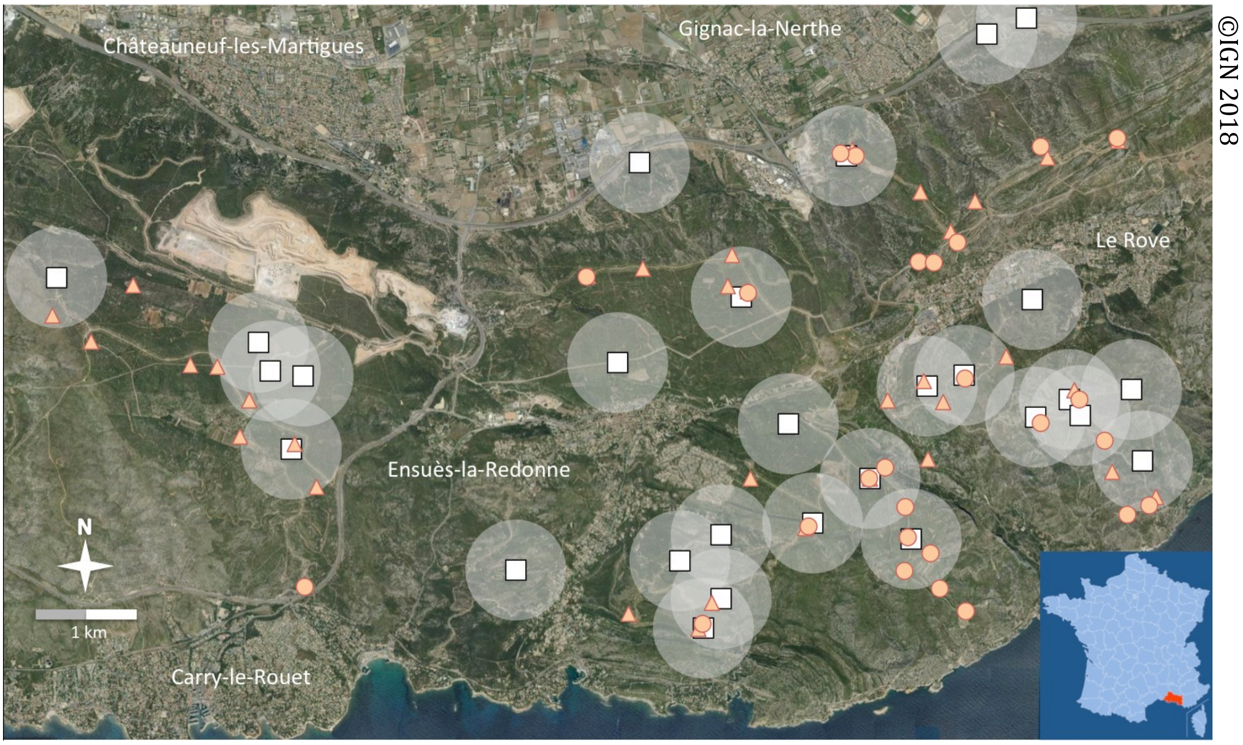

Supplementary Figure S3. Location of apiaries and sampling sites in the study area. Squares and shaded circles show apiary locations and the surrounding 500m radius areas. Coloured triangles and dots show the 2015 and 2016 sampling sites, respectively. Geographic data were obtained and mapped using the French Institut Géographique National open source databases and web mapping facilities (www.geoportail.gouv.fr, BD Ortho-photos imagery 2015, ©IGN 2018).

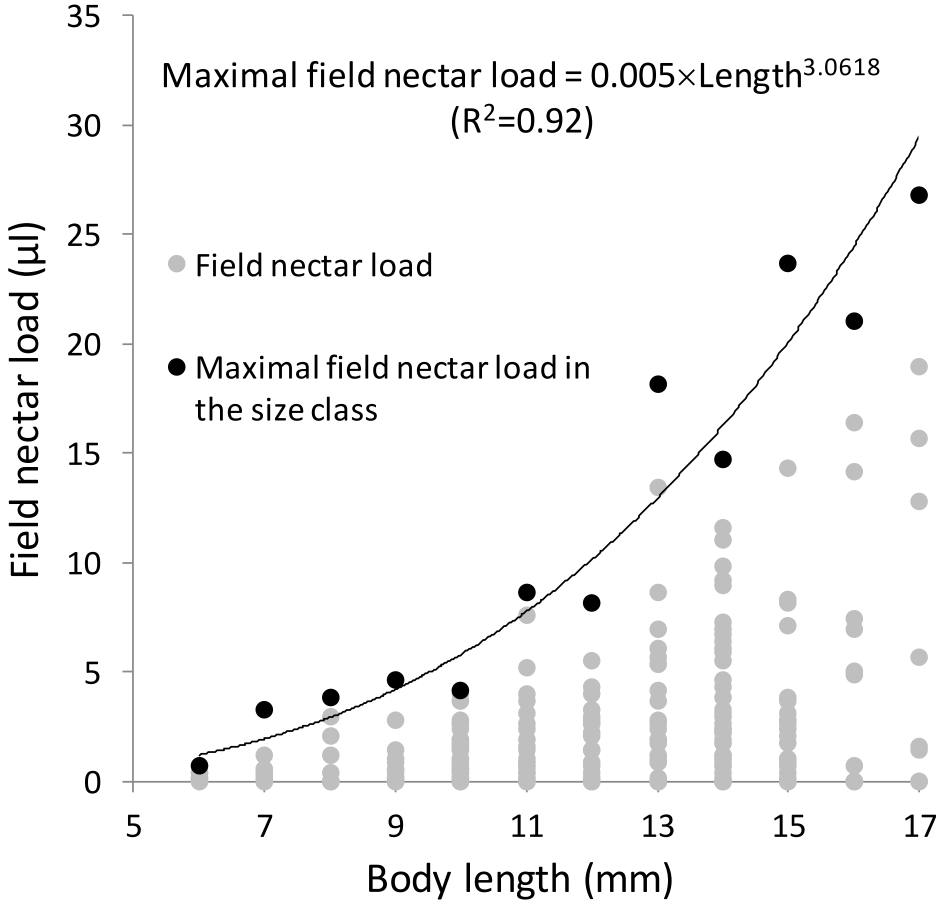

Supplementary Figure S4. **Allometric modelling of maximal field nectar load as a function of bee size (body length).** The curve indicates the predicted maximal load (µl) used to standardize observed nectar loads for a given body length. The length L, surface S and volume V of geometric bodies are not linearly related but scale on power laws of the form $S \propto L^2$ and $V \propto L^3$. In biology, power terms that are empirically derived from morphometric studies are often convergent with those theoretical laws, even though it is more common to focus on organ or organism's mass M instead of V, and to explore the corollaries $S \propto M^{2/3}$ and $L \propto M^{1/3}$ (e.g. [[49]] for insects). Here we expected a relationship of the form $V_{Nectar\ load} \propto L_{Boby}{}^3$, and empirically obtained a fairly close power term (3.06), which further supports the tractability of the approach.

Supplementary Table S1: Effect of increase colony density score on bee occurrence and foraging success. Wild bee occurrence in foraging surveys is better explained by the previous year's colony densities (inter-annual scale) than by current year densities (annual scale). Analogous statistics for distance effects are shown in Table 1.

Bee occurrence and foraging response variables*	Sample size (Nb of sites)	Intercept	Estimates†	Statistics	P-value (effect sign)	AIC weight (ω) ‡
Wild bees						
Wild bee foraging occurrence, inter-annual scale (Foraging intensity for 100 flowering volume units)	180 (60)	3.94 ± 0.52	-6.03 ± 0.26	z=-2.29	**0.022 (-)**	21.6%
Wild bee foraging occurrence, annual scale (Foraging intensity for 100 flowering volume units)	180 (60)	2.86 ± 0.36	0.08 ± 0.23	z=0.33	0.74	
Mean nectar foraging success (Standardized nectar crop content)	82 (35)	23.89 ± 5.65	-2.11 ± 3.85	t=-0.55	0.59	<1%
Mean pollen foraging success (Pollen load score)	78 (39)	30.47 ± 6.36	1.15 ± 4.67	t=0.25	0.81	
Body size, inter-annual scale (Body length, mm)	220 (44)	12.54 ± 0.48	-0.04 ± 0.30	t=-0.13	0.90	<1%
Body size, annual scale (Body length, mm)	220 (44)	12.58 ± 0.47	-0.08 ± 0.35	t=-0.22	0.82	<1%
Honeybees						
Mean nectar foraging success (Nectar crop content, µl)	144 (49)	8.45 ± 0.78	-1.96 ± 0.47	t=-4.15	**<0.001 (-)**	<1%
Mean pollen foraging success (Pollen load score)	106 (44)	2.36 ± 0.50	-0.21 ± 0.29	t=-0.72	0.47	<1%

* All models are LMMs, except wild bee foraging occurrence: Zero-Inflated GLMM (negative-binomial family distribution and log-link function); †Estimates stand for changes per 100 colony density units; ‡ AIC Weight of evidence in favour of the colony density effect being a better predictor than the apiary distance effect. The AIC weight ω is shown only when at least one of the two candidate predictors has a significant effect (see Table 1 for the apiary distance effect).

Supplementary Table S2: Preliminary bee species checklist for the study area and body size ranges (mm). Voucher specimens were collected during the standardized sampling sessions as well as in the course of additional prospections in the area. Whenever possible, additional body size data were obtained from the bee reference collection held at the *Abeilles et Environnement* INRA research unit in Avignon, France, in order to complete size samples to five specimens per species. Larger species with body size range exceeding the usual honeybee worker size (12-13 mm) are indicated in bold.

Species	Range (mm)
Andrena albopunctata	**14 - 17**
Andrena bicolor	9 - 10
Andrena combinata	9 - 11
Andrena hesperia	9 - 11
Andrena lagopus	9 - 11
Andrena mucida	11
Andrena nigroaenea	**11 - 15**
Andrena niveata	6 - 8
Andrena rhenana	10 - 11
Andrena senecionis	10 - 12
Andrena similis	9 - 11
Andrena vulpecula	8 - 9
Anthophora affinis	**17 - 19**
Anthophora atriceps	12 - 13
Anthophora dispar	**13 - 15**
Anthophora mucida	**19 - 21**
Anthophora plumipes	**14 - 15**
Bombus terrestris* ssp *lusitanicus	**12 - 24**
Bombus terrestris* ssp *terrestris	**12 - 24**
Colletes albomaculatus	**12 - 14**
Eucera caspica	**12 - 14**
Eucera hispana	**13 - 14**
Eucera nigrilabris	**16 - 18**
Halictus fulvipes	10 - 13
Halictus gemmeus	6 - 7
Halictus group *simplex*	8 - 11

Species	Range (mm)
Halictus scabiosae	**12 - 15**
Lasioglossum albocinctum	12 - 13
Lasioglossum bimaculatum	7 - 9
Lasioglossum griseolum	3 - 4
Lasioglossum malachurum	7 - 8
Lasioglossum maurusium	7
Lasioglossum pallens	7 - 8
Lasioglossum planulum	5 - 6
Lasioglossum subhirtum	7 - 9
Lasioglossum transitorium planulum	4 - 5
Megachile parietina	**18**
Nomada basalis	9 - 10
Nomada beaumonti	7 - 8
Nomada panzeri	6 - 8
Nomada succincta	11 - 12
Osmia aurulenta	8 - 11
Osmia bicornis	**11 - 15**
Osmia cornuta	**15 - 16**
Osmia rufohirta	8 - 12
Osmia tricornis	**17**
Osmia versicolor	6 - 8
Rhodanthidium septemdentatum	**11 - 14**
Rhodanthidium sticticum	**10 - 14**
Sphecodes albilabris	**11 - 14**
Xylocopa iris	**14 - 24**

Supplementary Table S3: Statistical power estimates for the probability to detect a medium effect size on bee competition metrics.

Competition metrics	Sample size	Statistical power*
Wild bee foraging occurrence	180	0.999
Wild bee mean nectar foraging success	82	0.934
Wild bee mean pollen foraging success	78	0.922
Honeybee mean nectar foraging success	144	0.996
Honeybee mean pollen foraging success	106	0.977

* Probability to detect a medium effect size (*sensu* [53]), defined as a Cohen's $f^2 = 0.15$, given sample size.

Supplementary Table S4. Complete pollen foraging success models including temporal interactions.

Model specifications*	Sample size (Nb of sites)	Intercept	Estimates (Statistics) Main effect	Time period†	Interaction‡
Wild bees					
Distance to nearest apiary	91 (41)	31.75 ± 3.75	-0.94 ± 4.99 (t=-0.18, P=0.85)	-7.75 ± 9.05 (t=-0.85, P=0.40)	-4.86 ± 28.19 (t=-0.17, P=0.86)
Colony density	91 (41)	30.13 ± 6.82	1.68 ± 4.91 (t=0.34, P=0.73)	-0.73 ± 18.80 (t=-0.03, P=0.97)	-6.48 ± 0.16 (t=-0.41, P=0.68)
Honeybees					
Distance to nearest apiary	129 (45)	1.59 ± 0.31	0.66 ± 0.22 (t=3.03, **P=0.003**)	0.59 ± 0.57 (t=1.04, P=0.30)	-0.98 ± 0.37 (t=-2.67, **P=0.009**)
Colony density	129 (45)	2.37 ± 0.50	-0.22 ± 0.28 (t=-0.75, P=0.45)	-0.96 ± 0.84 (t=-1.15, P=0.25)	0.50 ± 0.51 (t=0.99, P=0.32)

* All models are LMMs ; † Time period of the day, during the main daytime sampling (first to third time quartile) or during the late period (fourth quartile) ; ‡ Beekeeping × Time period statistical interaction.

Supplementary Table S5: Statistical details of selected or missing path coefficients from the path model.

Response	Predictor	Sample size (Nb of sites)	Estimates*	Statistic	P-value
Simple distance-mediated competition scenario : AICc = 74.89, AICc weight ω = 0.1% Test of deviation from conditional independence: C=19.22, df=12, P=0.083					
Selected path coefficients					
Pollen availability	Honeybee foraging occurrence	63 (21)	-0.33 ± 0.14	t=-2.29	**0.027**
Nectar availability	Honeybee foraging occurrence	100 (26)	-0.24 ± 0.08	t=-2.83	**0.006**
Honeybee foraging occurrence	Distance to nearest apiary	180 (60)	-0.26 ± 0.08	t=-3.11	**0.002**
Colony density score	Distance to nearest apiary	60 (60)	-0.34 ± 0.06	t=-6.26	**<0.001**
Missing path conditional coefficients					
Nectar availability	Distance to nearest apiary	100 (26)	0.91 ± 0.55	t=1.65	0.114
Pollen availability	Distance to nearest apiary	63 (21)	-0.03 ± 0.02	t=-1.32	0.204
Honeybee foraging occurrence	Colony density score	180 (60)	0.0021 ± 0.0010	t=2.19	**0.030**
Nectar availability	Colony density score	100 (26)	-0.0049 ± 0.0065	t=-0.76	0.456
Pollen availability	Colony density score	63 (21)	-0.0003 ± 0.0002	t=-1.13	0.275
Pollen availability	Nectar availability	63 (21)	-0.0011 ± 0.0034	t=-0.31	0.756
Joint effect distance-density competition scenario : AICc= 52.57, AICc weight ω =99.9% Test of deviation from conditional independence: C=8.75, df=10, P=0.556					
Selected path coefficients					
Pollen availability	Honeybee foraging occurrence	63 (21)	-0.33 ± 0.14	t=-2.29	**0.027**
Nectar availability	Honeybee foraging occurrence	100 (26)	-0.24 ± 0.08	t=-2.83	**0.006**
Honeybee foraging occurrence	Distance to nearest apiary	180 (60)	-0.26 ± 0.08	t=-3.19	**0.002**
Honeybee foraging occurrence	Colony density score (detrended from distance)	180 (60)	0.17 ± 0.08	t=2.19	**0.029**
Missing path conditional coefficients					
Nectar availability	Colony density score (detrended from distance)	100 (26)	-0.0012 ± 0.0070	t=-0.17	0.863
Pollen availability	Colony density score (detrended from distance)	63 (21)	-0.0001 ± 0.0003	t=-0.21	0.833
Nectar availability	Distance to nearest apiary	100 (26)	0.91 ± 0.55	t=1.65	0.114
Pollen availability	Distance to nearest apiary	63 (21)	-0.03 ± 0.02	t=-1.32	0.204
Pollen availability	Nectar availability	63 (21)	-0.0011 ± 0.0034	t=-0.31	0.756

* All estimates proceed from LMMs (see Methods) after variables were standardised in units of data range. Distances were log-corrected to reduce residual variance.
